# Plant growth enhancement and associated physiological responses are coregulated by ethylene and gibberellin in response to harpin protein Hpa1

**DOI:** 10.1007/s00425-013-2013-y

**Published:** 2014-01-07

**Authors:** Xiaojie Li, Bing Han, Manyu Xu, Liping Han, Yanying Zhao, Zhilan Liu, Hansong Dong, Chunling Zhang

**Affiliations:** 1 State Ministry of Education Key Laboratory of Integrated Management of Crop Pathogens and Insect Pests, Nanjing Agricultural University, Nanjing, 210095 China; 2 Tobacco Research Institute, Henan Provincial Academy of Agricultural Sciences, Xuchang, 461000 China

**Keywords:** Ethylene, Gibberellin, Hpa1, Plant growth enhancement, Signal transduction

## Abstract

The harpin protein Hpa1 produced by the bacterial blight pathogen of rice induces several growth-promoting responses in plants, activating the ethylene signaling pathway, increasing photosynthesis rates and *EXPANSIN* (*EXP*) gene expression levels, and thereby enhancing the vegetative growth. This study was attempted to analyze any mechanistic connections among the above and the role of gibberellin in these responses. Hpa1-induced growth enhancement was evaluated in *Arabidopsis*, tomato, and rice. And growth-promoting responses were determined mainly as an increase of chlorophyll a/b ratio, which indicates a potential elevation of photosynthesis rates, and enhancements of photosynthesis and *EXP* expression in the three plant species. In *Arabidopsis*, Hpa1-induced growth-promoting responses were partially compromised by a defect in ethylene perception or gibberellin biosynthesis. In tomato and rice, compromises of Hpa1-induced growth-promoting responses were caused by a pharmacological treatment with an ethylene perception inhibitor or a gibberellin biosynthesis inhibitor. In the three plant species, moreover, Hpa1-induced growth-promoting responses were significantly impaired, but not totally eliminated, by abolishing ethylene perception or gibberellin synthesis. However, simultaneous nullifications in both ethylene perception and gibberellin biosynthesis almost canceled the full effects of Hpa1 on plant growth, photosynthesis, and *EXP2* expression. Theses results suggest that ethylene and gibberellin coregulate Hpa1-induced plant growth enhancement and associated physiological and molecular responses.

**Electronic supplementary material:**

The online version of this article (doi:10.1007/s00425-013-2013-y) contains supplementary material, which is available to authorized users.

## Introduction

Harpin proteins produced by Gram-negative plant pathogenic bacteria have multifaceted functions in a variety of plant species and have drawn great attention in the last two decades (Wei et al. 1992; He et al. 1993; Kim and Beer 2000; Desikan et al. 2001; Alfano and Collmer 2004; Liu et al. 2006; Oh and Beer 2007; Chen et al. 2008a, b; Miao et al. 2010; Sang et al. 2012; Choi et al. 2013; Li et al. 2014). After ectopic expression in transgenic plants or external application to plants, harpins activate distinct signaling pathways to induce different beneficial effects (Chen et al. 2008a). In response to a harpin protein, the salicylic acid signaling pathway is activated to confer induced resistance against pathogens (Strobel et al. 1996; Dong et al. 1999; Peng et al. 2004; Sang et al. 2012), while abscisic acid signaling is triggered to regulate the induction of drought tolerance (Dong et al. 2005; Zhang et al. 2011a). In response to a harpin protein, moreover, the ethylene signaling pathway diverges downstream of the ethylene perception step into two distinct circuits: one leads to induced resistance against insect pests (Dong et al. 2004; Liu et al. 2011; Lü et al. 2013a; Zhang et al. 2011b), and the other results in plant growth enhancement (Dong et al. 2004; Ren et al. 2006a, b, 2008; Zhang et al. 2007; Chen et al. 2008a, b; Li et al. 2014). Overall, harpins have been recognized as multifunctional elicitors in plants.

One such harpin is Hpa1 secreted by *Xanthomonas oryzae*, an important bacterial pathogen of rice (Zhu et al. 2000; Peng et al. 2004; Liu et al. 2006). Hpa1 causes multiple beneficial effects in different plants (Peng et al. 2004; Liu et al. 2006; Ren et al. 2006a, b; Miao et al. 2010; Zhang et al. 2011a, b) and possesses all functions characterized early for its orthologs identified in different bacterial species (Wei et al. 1992; He et al. 1993; Strobel et al. 1996; Dong et al. 1999). This study pays a main attention to Hpa1-induced plant growth enhancement and associated growth-promoting responses at physiological and molecular levels. In different plants, Hpa1-induced growth-promoting responses have been shown as activated ethylene signaling pathway, promoted photosynthesis, and enhanced expression of *EXPANSIN* (*EXP*) genes, and these responses result in plant growth enhancement (Ren et al. 2006a, b; Wu et al. 2007; Chen et al. 2008a, b; Li et al. 2014). This study focuses on current questions in regard to any mechanistic connections among Hpa1-induced growth-promoting responses.

The first question concerns the functional relationship between the concomitant roles of Hpa1 in activating the ethylene signaling pathway and inducing the expression of *EXP* genes (Ren et al. 2006a, b; Wu et al. 2007; Chen et al. 2008a; Li et al. 2014). *EXP* genes encode expansin (EXP) proteins that have unique “loosening” effects on plant cell walls and form a super protein family in plants (Sampedro and Cosgrove 2005). Members in the family function in different aspects of plant growth and development, such as cell elongation and separation, cell wall disassembly, formation of leaf primordia, and morphogenesis of root hairs (Cosgrove 2001; Cho and Cosgrove 2002; Colmer et al. 2004; Sampedro and Cosgrove 2005; Zhao et al. 2012; Lü et al. 2013b). Among hundreds of EXPs identified in different plant species (http://www.bio.psu.edu/expansins/other_species.htm), EXP1 and EXP2 orthologs have been demonstrated to be required for the vegetative growth (Cox et al. 2004; Sloan et al. 2009; Wang et al. 2011). Especially, enhanced expression of *EXP1* and *EXP2* genes contributes to plant growth enhancement by Hpa1 either expressed in transgenic plants or applied to plants (Ren et al. 2006a, b; Wu et al. 2007; Chen et al. 2008a). In Hpa1-expressing transgenic rice (*Oryza sativa*) lines or rice plants treated with Hpa1, growth enhancement is correlated with enhanced expression of *OsEXP1* (Ren et al. 2006a, b; Chen et al. 2008a). In *Arabidopsis* *thaliana* plants treated with Hpa1, *AtEXP1* and *AtEXP2* are highly expressed compared to the steady-state level of the expression in control plants (Li et al. 2014). Similarly, tea (*Camellia sinensis*) orthologs of *EXP1*, *EXP10*, and *EXP18* are highly upregulated at the transcription level following the Hpa1 treatment (Wu et al. 2007). In these study cases, enhanced *EXP* expression correlates not only with enhanced vegetative growth, but also with increased output of the crop product (rice grain or tea leaves used as drinking material) (Ren et al. 2006a, b; Wu et al. 2007; Chen et al. 2008a, b). However, these studies only demonstrate the concomitance in ethylene signaling and *EXP* gene expression, but do not offer evidence for causal relationship between both responses. Indeed, there is as yet no study to establish any mechanistic or physiological connections between ethylene signaling and *EXP* expression although they coordinately respond to Hpa1.

The second question is regarding the roles of phytohormones, in addition to ethylene, in Hpa1-induced growth-promoting responses, especially *EX*P expression. Published studies demonstrate that auxin, ethylene, and gibberellin induce or inhibit *EXP* expression depending on the types of *EXP* orthologs and plant tissues or growth conditions. For instance, exogenous gibberellin induces the expression of *OsEXP4* to promote internode growth of deepwater rice (Cho and Kende 1997), but exogenous auxin represses *OsEXP1* expression in the plant under infection by *X. oryzae* (Ding et al. 2008). In *Arabidopsis*, auxin and ethylene induce *AtEXP7* and *AtEXP8* expression to regulate root hair initiation (Cho and Cosgrove 2002). Both hormones also induce the expression of *LeEXP1* and *LeEXP2* in ripening fruits of tomato (*Lycopersicon esculentum*) (Rose and Bennett 1999). In tomato plants, moreover, imbalance of auxin and ethylene is responsible for the inhibitory effect of the toxic compound cyanamide on root growth associated with *LeEXP9* and *LeEXP18* expression (Soltys et al. 2012). However, *LeEXP9* expression in the young stem requires gibberellin and contributes to cell elongation in the organ (Vogler et al. 2003). Apparently, *EXP* expression induced by ethylene or auxin is related to defense responses (Ding et al. 2008; Soltys et al. 2012; Zhao et al. 2012) and regulation of development or morphogenesis (Rose and Bennett 1999; Cho and Cosgrove 2002); whereas, gibberellin-induced *EXP* expression associates with the vegetative growth (Cho and Kende 1997; Vogler et al. 2003). Therefore, it is great of interest to study the possible role of gibberellin in Hpa1-induced plant growth enhancement and growth-promoting responses.

An additional question is related to photosynthesis as a fundamental basis of plant growth and Hpa1-induced plant growth enhancement (Li et al. 2014). In *Arabidopsis*, externally applied Hpa1 facilitates gas exchanges in mesophyll cells or mesophyll conductance to CO_2_, promotes CO_2_ transport from substomatal cavities into mesophyll cells, and therefore increases net photosynthesis/CO_2_ assimilation (*A*_N_) rates. This physiological response critically contributes to growth enhancement of the plant by Hpa1 (Li et al. 2014). Whether any hormone plays a role of Hpa1-promoted photosynthesis is unknown until now.

This study was attempted to elucidate a mechanistic connection between the functions of Hpa1 in activating the ethylene signaling pathway and in eliciting plant growth-promoting responses shown as changes in *A*_N_ rates and *EXP* expression. The role of gibberellin in the responses was also analyzed.

## Materials and methods

### Protein preparations

Proteins used in this study were prepared as previously described (Chen et al. 2008a). Proteins were produced by *Escherichia coli* cells transformed with the prokaryotic expression vector pET30a(+) (empty vector) or the recombinant vector containing an insert of the *X. oryzae* *hpa1* gene, which had been fused to the histidine (His) tag coding sequence. The empty vector preparation (EVP) and the Hpa1-His fusion protein preparation were purified by nickel chromatography and elution with imidazole. Highly purified Hpa1-His protein was collected from the 200-mM imidazole eluent and used in the experiments after the His tag was removed by treatment with the Novagen Enterokinase Cleavage Capture Kit (EMD Biosciences Inc., Darmstadt, Germany). The 200-mM imidazole eluent of the EVP preparation was used as a negative control in the experiments.

### Plant material, treatment, and growth scoring

*Arabidopsis* genotypes used in this study were the ecotype Col-0, its mutants *etr1*-*1* and *ga5*-*1* generated previously (seed stock numbers CS1092, CS237, and CS62; http://arabidopsis.org), and the *etr1*-*1 ga5*-*1* double mutant generated by pollinating *etr1*-*1* with pollens of *ga5*-*1*. Seeds of the tomato (*Lycopersicon esculentum*) variety Xiafen were purchased from a local market. Seeds of the rice (*Oryza sativa*) variety IRBB10 were maintained in this lab. Seeds were disinfested in a 1.5 % (w/v) solution of sodium hypochlorite for 10 min before sowing or treatment.

Highly purified Hpa1 was prepared as a 15 μg mL^−1^ aqueous solution amended with the surfactant Silwet-37 at 0.03 % (v/v). The mixture was used to immerse disinfected seeds. Treated *Arabidopsis* seeds were incubated on Murashige and Skoog agar medium in 10-cm square plates, and the treated tomato and rice seeds were incubated on wet filter papers in 9-cm Petri dishes. Alternatively, disinfected seeds were sown directly in 60-ml pots filled with potting soil (Dong et al. 2005) and 10-day-old seedlings were treated with the Hpa1 solution by spraying over plant tops. In the experimental control group, highly pure water and an aqueous solution of EVP were amended with 0.03 % Silwet-37 and applied separately to seeds or plants similarly as the application of Hpa1. Plants in pots and on plates were grown in environmentally controlled chambers with a 12-h day (200 μmol photons m^−2^ s^−1^ and 24 °C) and 12-h night (20 °C) cycle. Root length of plants grown on the medium and fresh weight of plants grown in pots were surveyed.

### Physiological studies

Leaf content of total nitrogen was determined by a previously described method (Lang 1958). Concentrations of chlorophyll a and b were analyzed with Single-Photon Avalanche Diode (SPAD) meter (SPAD-502, Minota, Tokyo, Japan) and quantified as mg cm^−2^ leaf (Kourill et al. 1999; Yang et al. 2003). Leaf photosynthesis was analyzed with the Li-6400XT portable photosynthesis system and equipped leaf chambers (LI-COR, Inc., Lincoln, NE, USA). Detailed measurements were accomplished by following the manufacturer’s instructions and experimental procedures described previously (Flexas et al. 2007; Heckwolf et al. 2011). Gas concentrations at the inlet and outlet of leaf chambers were monitored by the non-dispersive infrared gas analyzer installed in the system. Instantaneous gas exchange measurements were performed several times during the experiments at saturating light (photosynthetically active photon flux density of 1,500 μmol m^−2^ s^−1^) and the chamber CO_2_ concentration of 400 μM M^−1^ air. Values of *A*_N_ were recorded by the S-501 digital monitor integrated into the system.

Endogenous ethylene and gibberellin concentrations were analyzed. The production of ethylene in plants was determined by a previously described protocol (Dong et al. 2004). Gibberellin content was analyzed as previously described (Foster et al. 1994). Gibberellin isoforms were extracted from growing stems of tomato and rice or the aerial parts of *Arabidopsis*, purified using a combination of preparatory column chromatography, and analyzed with reverse-phase high-performance liquid chromatography. Deuterated internal standards were added, 20 ng of [17,17-^2^H_2_]GA12 and 25 ng [17,17-^2^H_2_]GA1, -GA8, -GA19, -GA20, -GA44, and -GA53, respectively. Tritiated standards (1,500 Bq [1,2-^3^H_2_]GA1 and [1,2-^3^H_2_]GA4, respectively) were also added to the combined extract to monitor recovery through the purification procedure. Gas was quantified using GC–MS selected ion monitoring by calculating the area ratio of endogenous gibberellin to the deuterated standard gibberellin that had been added during the extraction step, and the contribution from the deuterated standard to the nondeuterated gibberellin was corrected (Beall et al. 1991). The concentration of total gibberellin (a sum of different isoforms) was quantified in contrast to fresh weight of plant tissues used in gibberellin isolation.

### Gene expression analysis

Gene expression was analyzed by reverse transcriptase-polymerase chain reaction (RT-PCR) and real-time RT-PCR (Chen et al. 2008a; Liu et al. 2011). The constitutively expressed *EF1α* and *Actin* genes were used as references. Genes tested and specific primers are provided in Supplementary Table S1. In real-time RT-PCR analysis, no template control was included to verify amounts of gene transcripts. The amount of a gene transcript was quantified relative to *EF1α*.

### Pharmacological study

Chemicals used in pharmacological study were the gibberellin biosynthesis inhibitor paclobutrazol (PBZ; Shanghai Chemical Reagent Co., Shanghai, China) and the ethylene signaling inhibitor 1-methylcyclopropene (1-MCP; Lytone Enterprise Inc., Nanjing Agency). PBZ was used at a physiologically active concentration (1 μM; Li et al. 2014) together with the Hpa1 solution to treat seeds or plants. 1-MCP was applied at a physiologically active concentration (0.225 μl L^−1^) to plants as previously described (Zhang et al. 2007; Ren et al. 2008). 1-MCP was supplied as 1.1 g water-volatilizable tablets containing 0.18 % active ingredient, and each tablet was determined by the supplier to release 0.9 μL L^−1^ gaseous 1-MCP in a 1-cm^3^ space (equivalent to 0.075 μL L^−1^ gaseous 1-MCP in a 12-cm^3^ space) at 20 °C and 70 % humidity. The plant treatment with 1-MCP was performed at the dark cycle (20 °C and 70 % humidity) in the chamber. Immediately before treatment, three tablets of the 1-MCP product were placed in a small beaker to release 1-MCP gas into plants grown in pots. The pots were placed together with the beaker in a 12-cm^3^ glass box while the box was sealed immediately after treatment. Therefore, the final concentration of gaseous 1-MCP was 0.225 μL L^−1^ applied to plants under this experiment condition. Plant treatments with 1-MCP and PBZ were maintained for 6 h, and subsequently, photosynthesis and *EXP* expression in leaves and plant growth were determined.

### Statistical analysis

All experiments were carried out at least three times with similar results. Quantitative data were analyzed with the IBM SPSS19.0 software package (IBM Corporation, Armonk, NY, USA; http://www-01.ibm.com/software/analytics/spss/) according to instructions in a text book that describes in detail the analysis methods using IBM SPSS19.0 (Shi 2012). Homogeneity-of-variance in data from *Arabidopsis*, tomato, or rice plants treated differently was determined by Levene test, and formal distribution pattern of the data was confirmed by Kolmogorov–Smirnov test and P–P plots. Then, data were analyzed by the ANOVA method along with Fisher’s least significant difference (LSD).

## Results

### Hpa1-induced plant growth enhancement

**Fig. 1:**
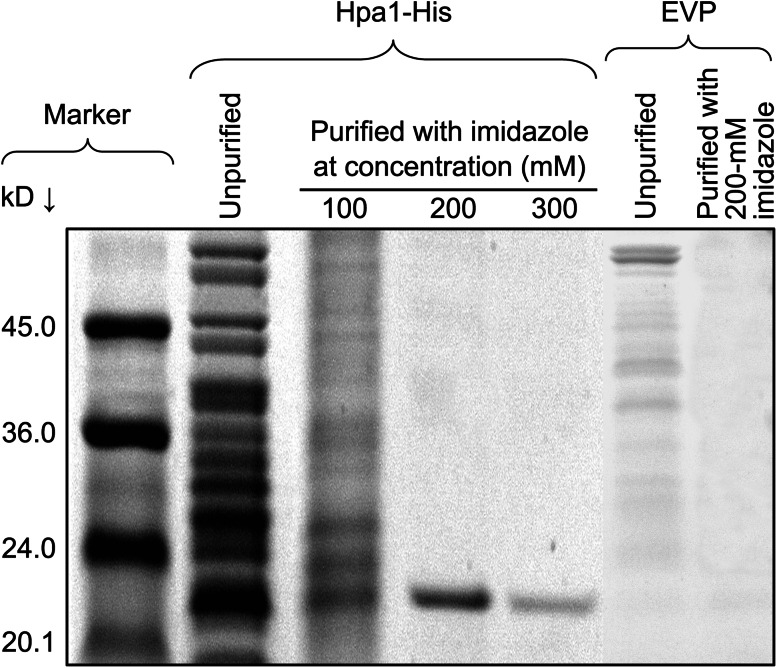
Protein electrophoresis analysis. The Hpa1-His fusion protein was produced through a recombinant prokaryotic vector and analyzed in comparison with the empty vector preparation (EVP) that did not contain Hpa1-His. Protein samples without purification were analyzed directly by electrophoresis on the tricine-sodium dodecylsulphate-plolyacrylamide gel. Alternatively, protein samples were bound to nickel-polystyrene beads, eluted with the indicated concentrations of imidazole, and then analyzed by electrophoresis. Protein bands were visualized by gel staining with Coomassie G-250. Molecular makers are indicated. The Hpa1-His fusion protein from the 200-mM imidazole eluent was treated with an enterokinase cleavage capture reagent to remove the His tag and only Hpa1 was used in plant treatment. The 200-mM imidazole eluent of the EVP preparation was used as a negative control

For the sake of biological consistency and representativeness, we analyzed Hpa1-induced growth-promoting responses by parallel experiments performed on *Arabidopsis* as a biological model, tomato in which harpin-induced plant growth enhancement was first observed (Kim and Beer 2000), and rice as it is the host of the Hpa1-producing pathogen (Zhu et al. 2000). An aqueous solution of highly purified Hpa1 protein (Fig. 1) was used to immerse seeds or treat plants by spraying over plant tops. Seeds and plants were treated similarly with pure water or an aqueous solution of EVP prepared similarly as for Hpa1 (Fig. 1) in the experimental control group.

**Fig. 2:**
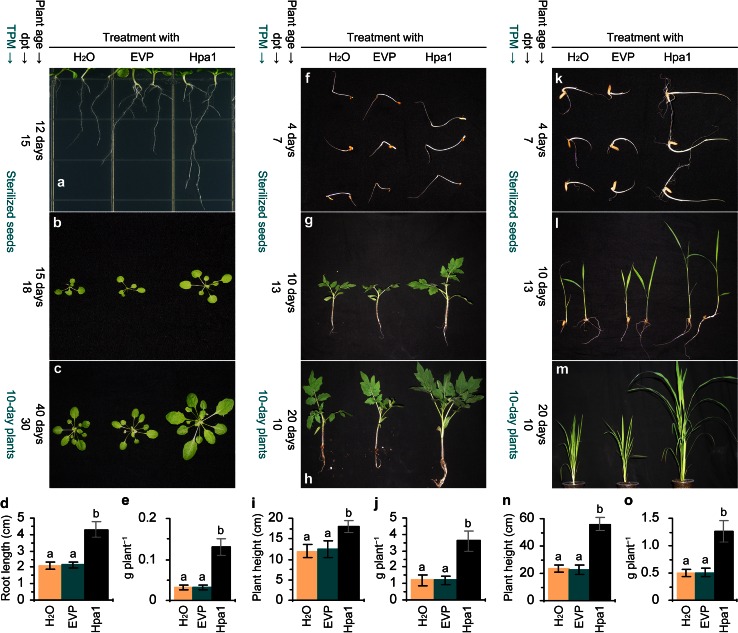
Hpa1-induced growth enhancement of *Arabidopsis* (**a**–**e**), tomato (**f**–**h**), and rice (**k**–**m**). Pure water and aqueous EVP and Hpa1 solutions were used separately to treat seeds by immersion or treat plants by spraying over plant tops. “TPM” refers to treated plant material, either sterilized seeds or 10-day-old seedlings; “dpt” denotes days posttreatment; “plant age” is the count of days after seed germination. Results shown in every treatment and plant species combination represent four experimental repeats and a total of 80 plants (20 plants per repeat). **a** *Arabidopsis* seedlings photographed at day 15 after treated seeds were inoculated on the medium. **b** *Arabidopsis* seedlings photographed at day 18 after treated seeds were inoculated on the potting soil. **c** Forty-day-old *Arabidopsis* plants from pots and photographed at day 30 after treatment. **d** Root length of *Arabidopsis* seedlings from **a**. **e** Fresh weight of *Arabidopsis* plants from **d**. Quantitative data shown in bar graphs are mean values ± standard deviation (SD) bars. **f**, **k** Tomato (**f**) and rice (**k**) seedlings photographed at day 7 after treated seeds were incubated on wet filter papers. **g**, **l** Tomato (**g**) and rice (**l**) seedlings photographed at day 13 after treated seeds were incubated in potting soil. **i**, **j**, **n**, **o** Biomass of tomato (**i**, **j**) from **h** and rice (**n**, **o**) from **m**. Data shown in *bar* graphs (*bottom panels*) are mean values ± standard deviation *bars*, and *different letters* on SD *bars* indicate significant differences analyzed by two-tailed ANOVA and Fisher’s least significant difference (LSD) test (*P* < 0.01)

In *Arabidopsis*, Hpa1-induced growth enhancement was observed with seedlings grown on the medium (Fig. 2a) or in the potting soil (Fig. 2b) following seed treatment. The application of Hpa1 to 10-day-old plants in pots also enhanced plant growth in the subsequent 30 days (Fig. 2c). Growth enhancement was quantified as an increase of root growth on the medium (Fig. 2d) and an increase of fresh weight of plants in pots (Fig. 2e). In tomato (Fig. 2f–j) and rice (Fig. 2k–o), Hpa1-induced growth enhancement was observed after treated seeds were incubated on filter papers (Fig. 2f, k) or sown in the potting soil (Fig. 2g, l). The application of Hpa1 to 10-day-old plants in pots also enhanced plant growth in the subsequent 10 days (Fig. 1h, m). Growth enhancement of both tomato and rice was quantified as higher values of height (Fig. 2i, n) and fresh weight (Fig. 2j, o) of 20-day-old plants, tested at day 10 after plant top treatment.

In all plants, differences in growth extents were significant (*P* < 0.01) between the Hpa1 treatment and the treatment with water or EVP. In *Arabidopsis*, the Hpa1 treatment caused a 1.9-fold increase in root length of seedlings grown on the medium (Fig. 2d) and a 1.7-fold increase in fresh weight of plants grown in pots (Fig. 2e). In tomato, the multiple of increase in plant height was 1.2 (Fig. 2i) and the multiple of increase in fresh plant weight were 3 (Fig. 2j) following the plant treatment with Hpa1 compared to EVP or water. In rice, the Hpa1 treatment caused a 2.4-fold increase in plant height (Fig. 2n) and a 2.5-fold increase fresh plant weight (Fig. 2o).

### Hpa1-induced physiological responses related to plant growth

To reveal the physiological basis of Hpa1-induced plant growth enhancement, we analyzed the effects of Hpa1 on nitrogen and chlorophyll concentrations and *A*_N_ rates in leaves of *Arabidopsis*, tomato, and rice. Among the many influencing factors, nitrogen is the most limiting resource for plant growth, and about 80 % nitrogen in leaves is deposited in chloroplast, an organ functioning as a center of biosynthesis and productivity. Measurement values of total nitrogen content in Hpa1-treated *Arabidopsis* plants was 1.89 ± 0.21 mg g^−1^ fresh leaf, counting for a 43.2 % increase compared to the measurement value of 1.32 ± 0.17 mg g^−1^ fresh leaf in control plants. Total nitrogen content in leaves of Hpa1-treated tomato plants was 5.54 ± 0.29 mg g^−1^ fresh leaf, and this value was 36.5 % greater than that (4.06 ± 0.43 mg g^−1^ fresh leaf) in control plants. Similarly, values of total nitrogen content in Hpa1-treated rice plants were 7.21 ± 0.36 mg g^−1^ fresh leaf, counting for a 25.4 % increase compared to the measurement value of 5.75 ± 0.21 mg g^−1^ fresh leaf in control plants. In all plants, nitrogen concentrations were significantly (*P* < 0.01) increased by the treatment with Hpa1 compared to water or EVP.

**Fig. 3:**
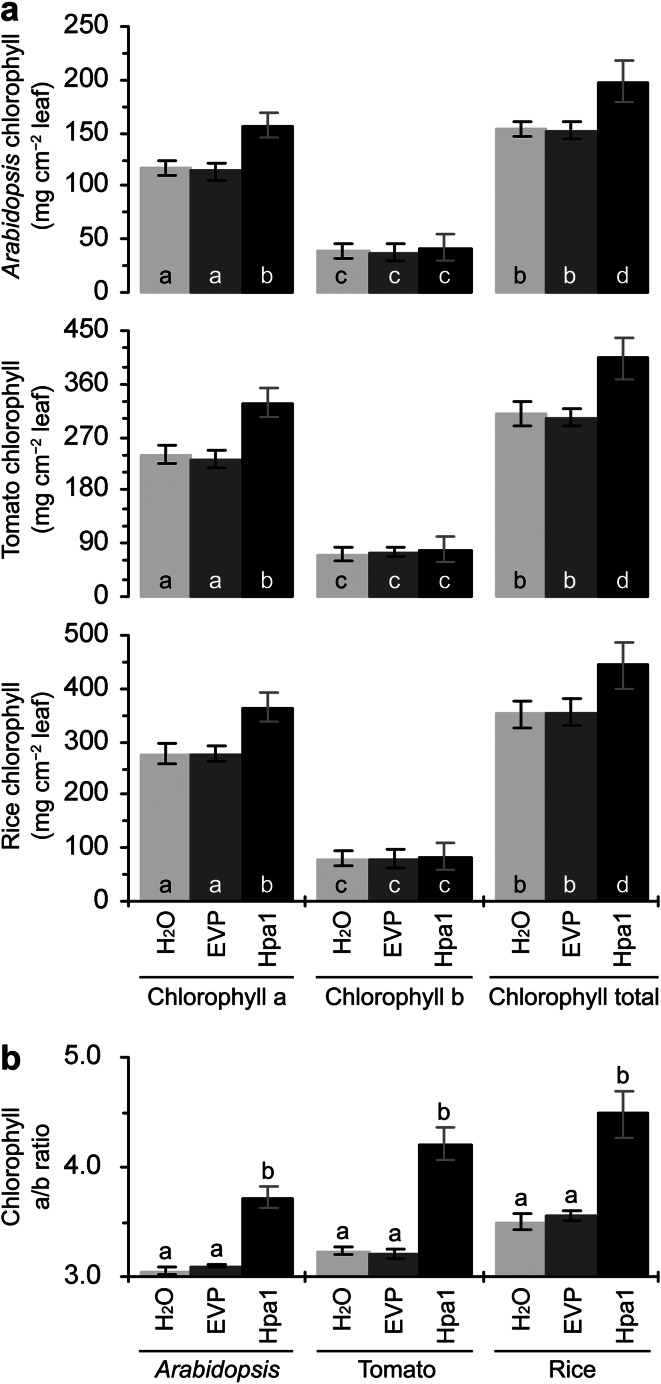
The effects of Hpa1 on chlorophyll a and b concentrations (**a**) and chlorophyll a/b ratio (**b**) in leaves of *Arabidopsis*, tomato, and rice. Ten-day-old plants grown in pots were treated by spraying over tops separately with an aqueous Hpa1 solution and with pure water or an aqueous EVP solution in the experimental control group. Five days later, chlorophyll measurements were performed on the second youngest leaves. Data are shown as mean values ± SD from three experimental repeats (15 plants per repeat). *Different letters* in *bar* graphs indicate significant differences by two-tailed ANOVA and LSD test (*P* < 0.01)

We determined the chlorophyll level since elevation in leaf nitrogen from a moderate level of basal content may promote photosynthesis, which depends on the function of chlorophylls and correlates with leaf chlorophyll content to a certain extent (Yang et al. 2003). In *Arabidopsis* and tomato plants, levels of total chlorophylls and chlorophyll a were increased significantly (*P* < 0.01) by the treatment with Hpa1 compared to water or EVP, and the Hpa1 treatment caused a slight increase in the content of chlorophyll b (Fig. 3a). In the three plants, moreover, the Hpa1 treatment significantly (*P* < 0.01) increased the chlorophyll a/b ratio (Fig. 3b). Because the chlorophyll a/b ratio correlates with functional maturity of the photosynthetic apparatus and with the photosynthetic activity (Kourill et al. 1999), the observed increase of the ratio indicates that photosynthesis may be promoted by the Hpa1 treatment.

**Fig. 4:**
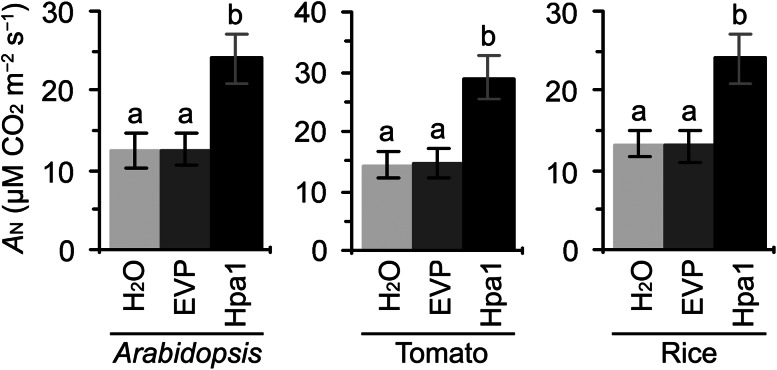
The effect of Hpa1 on photosynthesis in leaves of *Arabidopsis*, tomato, and rice. Ten-day-old plants grown in pots were treated by spraying over tops separately with an aqueous Hpa1 solution and with pure water or an aqueous EVP solution. Five days later, measurements of net photosynthesis (*A*_N_) rates were performed on the second youngest leaves. Data are shown as mean values ± SD from three experimental repeats (15 plants per repeat). *Different letters* on SD *bars* indicate significant differences by two-tailed ANOVA and LSD test (*P* < 0.01)

This hypothesis was confirmed by measurements of *A*_N_ rates. Values of *A*_N_ in leaves of *Arabidopsis*, tomato, and rice plants were significantly (*P* < 0.01) increased by the Hpa1 treatment compared to control (Fig. 4). Multiple values of Hpa1-increased *A*_N_ rates were 1.9 in *Arabidopsis*, 1.7 in tomato, and 2.0 in rice. This analysis and analyses of nitrogen and chlorophyll together suggest that increased photosynthesis is an important physiological change associated with Hpa1-induced plant growth enhancement.

### Hpa1-enhanced expression of *EXP* genes required for plant growth

**Fig. 5:**
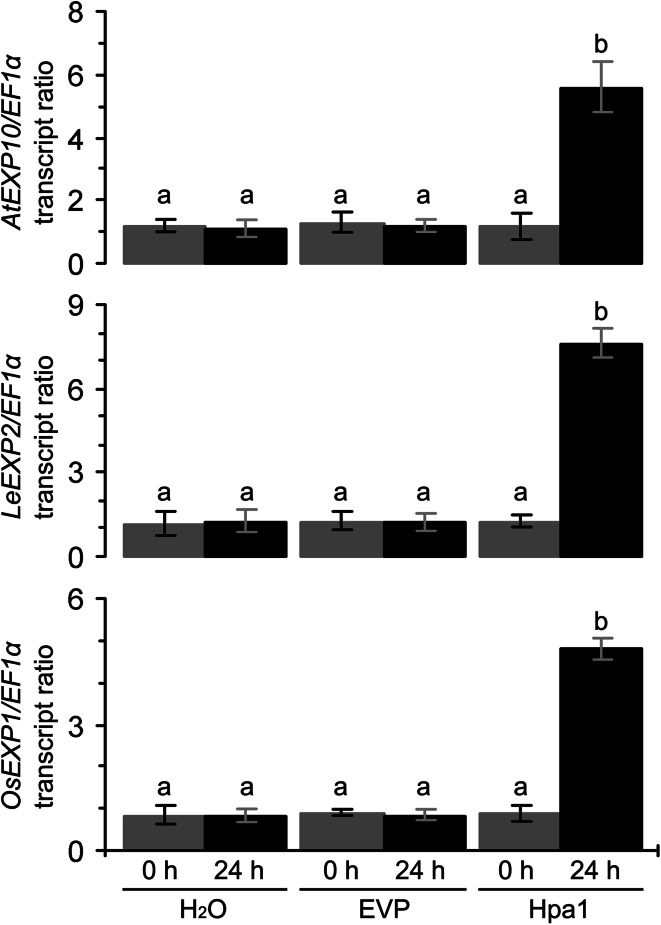
The effect of Hpa1 on the expression of *EXP* genes in *Arabidopsis*, tomato, and rice. Ten-day-old plants grown in pots were treated by spraying over tops separately with an aqueous Hpa1 solution and with pure water or an aqueous EVP solution. Total RNA was isolated from the second youngest leaves and analyzed by real-time reverse transcriptase-polymerase chain reaction (RT-PCR). Amounts of *EXP* transcripts were quantified in relative to that of *EF1α*, a constitutively expressed gene used as a reference. Data are shown as mean values ± SD from three experimental repeats (15 plants per repeat). *Different letters* on SD *bars* indicate significant differences by two-tailed ANOVA and LSD test (*P* < 0.01)

To reveal the molecular basis of Hpa1-induced plant growth enhancement, we determined *EXP* gene expression in Hpa1-treated and control plants. Based on published sequences, we studied five and three *EXP* genes of *Arabidopsis* and tomato, respectively. These genes exhibited steady-state expression in control plants but their expression was highly enhanced by Hpa1 and increased with time in 4 days after the Hpa1 treatment (Supplementary Fig. S1). *AtEXP10* and *LeEXP2* were expressed at the most substantial extents in *Arabidopsis* and tomato, respectively. The expression of both genes reached the highest levels in 24 h compared to other time points in 4 days after the Hpa1 treatment (Supplementary Fig. S1). In 24 h, levels of *AtEXP10* and *LeEXP2* expression in Hpa1-treated plants were increased significantly (*P* < 0.01) and were approximately five times of the steady-state expression level, which was detected in control plants or in Hpa1-treated plants immediately after treatment (Fig. 5). In accordance with our previous studies (Ren et al. 2006a, b; Chen et al. 2008a), moreover, enhanced expression of *OsEXP1* (Fig. 5 and Supplementary Fig. S1) was found to be associated with growth enhancement (Fig. 1g–i) of rice plants following treatment with Hpa1. Therefore, enhanced expression of *AtEXP10*, *LeEXP2*, and *OsEXP1* were used as a molecular marker of Hpa1-induced plant growth enhancement.

### Hpa1-enhanced production of ethylene and gibberellin in plants

**Fig. 6:**
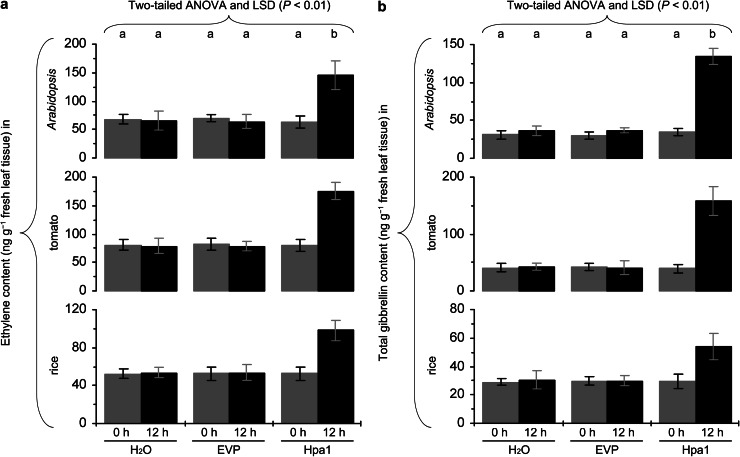
The effects of Hpa1 on concentrations of phytohormones ethylene (**a**) and gibberellin (**b**) in *Arabidopsis*, tomato, and rice plants. Ten-day-old plants grown in pots were treated by spraying over tops separately with an aqueous Hpa1 solution and with pure water or an aqueous EVP solution. Concentrations of both hormones were determined at the indicated time points. Data are shown as mean values ± SD from three experimental repeats (15 plants per repeat). *Different letters* on tops of *bar* graphs indicate significant differences by the statistical analysis

In order to correlate Hpa1-induced plant growth enhancement with the production of ethylene and gibberellin, we determined the amount of ethylene released from *Arabidopsis*, tomato, and rice plants and the production of gibberellin in three plants following treatment with Hpa1 vs. water and EVP. Results are provided in Fig. 6. In water-treated or EVP-treated control plants, the amount of ethylene released and the endogenous concentration of total gibberellin changed little in 12 h. By contrast, the Hpa1 treatment caused significant (*P* < 0.01) increases in ethylene release and total gibberellin content in *Arabidopsis*, tomato, and rice. In the three plants treated with Hpa1, ethylene was increased by more than twice and gibberellin was increased approximately by 4 folds in contrast to the hormone levels in control plants.

### The activation of plant ethylene signaling by Hpa1

To address whether ethylene signaling is involved in Hpa1-induced plant growth enhancement, we analyzed the expression of ethylene-signaling genes (*ETR1* and *EIN5*) and ethylene-responsive genes (*PDF1.2* and *PR*-*3b*) in *Arabidopsis* plants following treatment with water, EVP, and Hpa1, respectively. The four genes represent three major steps of ethylene signal transduction. First, the *ETR1* gene encodes ethylene receptor protein ETR1 essential for perception of the ethylene signal and its subsequent transduction to the expression of ethylene-responsive genes (Gamble et al. 2002). *ETR1* is also required for *Arabidopsis* growth enhancement by the HrpN_Ea_ harpin from *Erwinia amylovora*, the pathogen that causes fire blight in rosaceous plants (Dong et al. 2004). In Hpa1-treated plants, *ETR1* was expressed 6 h after treatment and expression level was increased with time in the subsequent 90 h (Supplementary Fig. S2). Inversely, *ETR1* expression was not detected in control plants. Second, the *EIN5* gene encodes an exoribonuclease (Olmedo et al. 2006; Potuschak et al. 2006), which is an essential component of the ethylene signaling pathway and regulates plant growth enhancement by HrpN_Ea_ (Dong et al. 2004). The *EIN5* gene had a steady-state level of expression in control plants; whereas, its expression level was enhanced in Hpa1-treated plants and increased with time in 6–96 h after treatment (Supplementary Fig. S2). Third, the *PDF1.2* and *PR*-*3b* genes are molecular markers of the ethylene signaling pathway (Pieterse et al. 1998; Penninckx et al. 1998). Both genes were expressed only in Hpa1-treated plants (but not in water-treated or EVP-treated control plants) and accumulated transcripts detectable by RT-PCR at hour 12 after the Hpa1 treatment and increased expression levels in next 84 h (Supplementary Fig. S2). These analyses indicate that the ethylene signaling pathway is activated by the Hpa1 treatment.

### The role of ethylene in Hpa1-enhanced photosynthesis, *EXP* expression, and plant growth

**Fig. 7:**
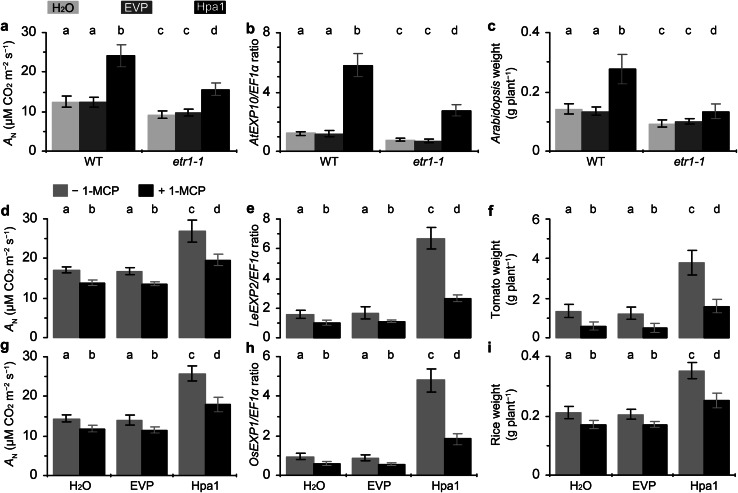
The inhibitory effects of genetic or chemical blocking in ethylene perception on Hpa1-induced enhancements of photosynthesis, *EXP* expression, and plant growth. **a**–**c** The *Arabidopsis* mutant *etr1*-*1*, which has a defect in the ethylene receptor ETR1 and fails to sense and transmit the ethylene signal, was tested together with the wild-type (WT) plant in the experiments. Ten-day-old plants were treated separately with an aqueous Hpa1 solution and with pure water or an aqueous EVP solution. **d**–**i** The ethylene signaling inhibitor 1-methylcyclopropene (1-MCP) was used to block ethylene signal transduction in tomato (**d**–**f**) and rice (**g**–**i**) plants. Ten-day-old plants were treated with water or aqueous EVP and Hpa1 solutions in the presence of 1-MCP (+1-MCP) and the absence of 1-MCP (–1-MCP). **a**–**i** Five days after plant treatment, analyses of *A*_N_ rates and *EXP* expression were performed on the second youngest leaves. Fresh weight of plants was scored 10 days after treatment. Data are shown as mean values ± SD from three experimental repeats (15 plants per repeat). *Different letters* on tops of *bar* graphs indicate significant differences by the statistical analysis

To elucidate whether ethylene signaling plays a role in Hpa1-enhanced photosynthesis, *EXP* expression, and plant growth, we first analyzed these responses in the wild-type (WT) plant of *Arabidopsis* and its mutant *etr1*-*1*, which has a defect in ETR1 and fails to sense and transmit the ethylene signal (Schaller and Bleecker 1995; Gamble et al. 2002). We found that photosynthesis was diminished in *etr1*-*1* compared to WT (Fig. 7a). The mutant was impaired in normal photosynthesis as indicated by a lower value of the *A*_N_ rate relative to that of WT detected in control (treatment with water or EVP). The mutant was further impaired in the promoting effect of Hpa1 on photosynthesis, and Hpa1-increased percentage of the *A*_N_ rate over the rate in control was 92 % in WT and 48 % in *etr1*-*1*. In the mutant, *AtEXP10* expression and the enhancement by Hpa1 were compromised concomitantly (Fig. 7b). Hpa1-increased part of *AtEXP10* expression was diminished by 47 %, but not canceled totally. In *etr1*-*1*, moreover, normal growth and Hpa1-induced growth enhancement were both impaired (Fig. 7c). The Hpa1 treatment caused increases of fresh plant weight by 93 % in WT and 53 % in *etr1*-*1*, indicating that Hpa1-induced growth enhancement was diminished but not totally canceled by the genetic blocking of ethylene perception in the mutant.

We treated tomato (Fig. 7d–f) and rice (Fig. 7g–i) plants with the ethylene signaling inhibitor 1-MCP, which was used in an aqueous Hpa1 solution and with pure water or EVP in control. We found that the pharmacological effects of 1-MCP resembled the genetic effects of *etr1*-*1* on photosynthesis (Fig. 7d, g), *EXP* expression (Fig. 7e, h), plant growth (Fig. 7f, i), and enhancements of these events by Hpa1 (Fig. 7d–i). The application of 1-MCP to inhibit ethylene signaling only diminished, but did not completely eliminate, Hpa1-induced plant growth enhancement and associated enhancements in photosynthesis and *EXP* expression (Fig. 7d–i). This result and the analysis of *etr1*-*1* together indicate that ethylene signaling plays a partial role in regulating Hpa1-enhanced plant growth and the associated responses.

### The role of gibberellin in Hpa1-enhanced photosynthesis, *EXP* expression, and plant growth

**Fig. 8:**
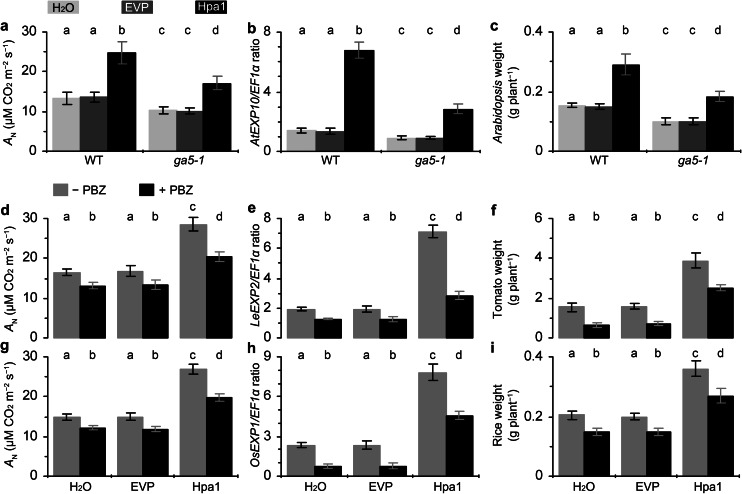
The inhibitory effects of genetic or chemical blocking in gibberellin biosynthesis on Hpa1-induced enhancements of photosynthesis, *EXP* expression, and plant growth. **a**–**c** The *Arabidopsis* mutant *ga5*-*1*, which has a defect in gibberellin biosynthesis, was tested together with the WT plant in the experiments. Ten-day-old plants were treated separately with an aqueous Hpa1 solution and with water or an aqueous EVP solution. **d**–**i** The gibberellin biosynthesis inhibitor PBZ was used to inhibit gibberellin biosynthesis in tomato (**d**–**f**) and rice (**g**–**i**) plants. Ten-day-old plants were treated separately with Hpa1 and Silwet-37 in the presence of PBZ (+ PBZ) and the absence of PBZ (–PBZ). **a**–**i** Five days after plant treatment, analyses of *A*_N_ rates and *EXP* expression were performed on the second youngest leaves. Fresh weight of plants was scored 10 days after treatment. Data are shown as mean values ± SD from three experimental repeats (15 plants per repeat). *Different letters* on tops of *bar* graphs indicate significant differences by two-tailed ANOVA and LSD test (*P* < 0.01)

To elucidate the role of gibberellin in Hpa1-enhanced photosynthesis, *EXP* expression, and plant growth, the *Arabidopsis* *ga5*-*1* mutant, which is defective in gibberellin biosynthesis (Xu et al. 1995; Sponsel et al. 1997), was tested together with the WT plant in responses to the Hpa1 treatment. We found that photosynthesis was diminished in *ga5*-*1* compared to WT (Fig. 8a). The mutant was impaired in normal photosynthesis as indicated by a lower value of the *A*_N_ rate relative to that of WT detected in control (treatment with water or EVP). The mutant was further impaired in the promoting effect of Hpa1 on photosynthesis, and Hpa1-increased percentage of the *A*_N_ rate over the rat in control was 98 % in WT and 53 % in *ga5*-*1*. In the mutant, *AtEXP10* expression and the enhancement by Hpa1 were compromised concomitantly (Fig. 8b). Hpa1-increased part of *AtEXP10* expression was diminished by 47 % but not canceled totally. In *ga5*-*1*, moreover, normal growth and Hpa1-induced growth enhancement were both impaired (Fig. 8c). The Hpa1 treatment caused increases of fresh plant weight by 96 % in WT and 48 % in *etr1*-*1*, indicating that Hpa1-induced growth enhancement was diminished but not totally canceled in the mutant.

We then investigated responses of tomato (Fig. 8d–f) and rice (Fig. 8g–i) plants to the gibberellin biosynthesis inhibitor PBZ, which was applied together with Hpa1, water, or EVP. We found that the pharmacological effects of PBZ resembled the genetic effects of *ga5*-*1* on photosynthesis (Fig. 8d, g), *EXP* expression (Fig. 8e, h), and plant growth (Fig. 8f, i), and enhancements of these events by Hpa1 (Fig. 8d–i). Therefore, the application of PBZ to inhibit gibberellin synthesis only diminished, but did not completely eliminate, Hpa1-induced plant growth enhancement, and associated enhancements in photosynthesis and *EXP* expression. This result is consistent with the analysis of *ga5*-*1*, indicating that Hpa1-enhanced plant growth and the associated responses are partially regulated by gibberellin.

### The interactive role of ethylene and gibberellin in Hpa1-enhanced photosynthesis, *EXP* expression, and plant growth

**Fig. 9:**
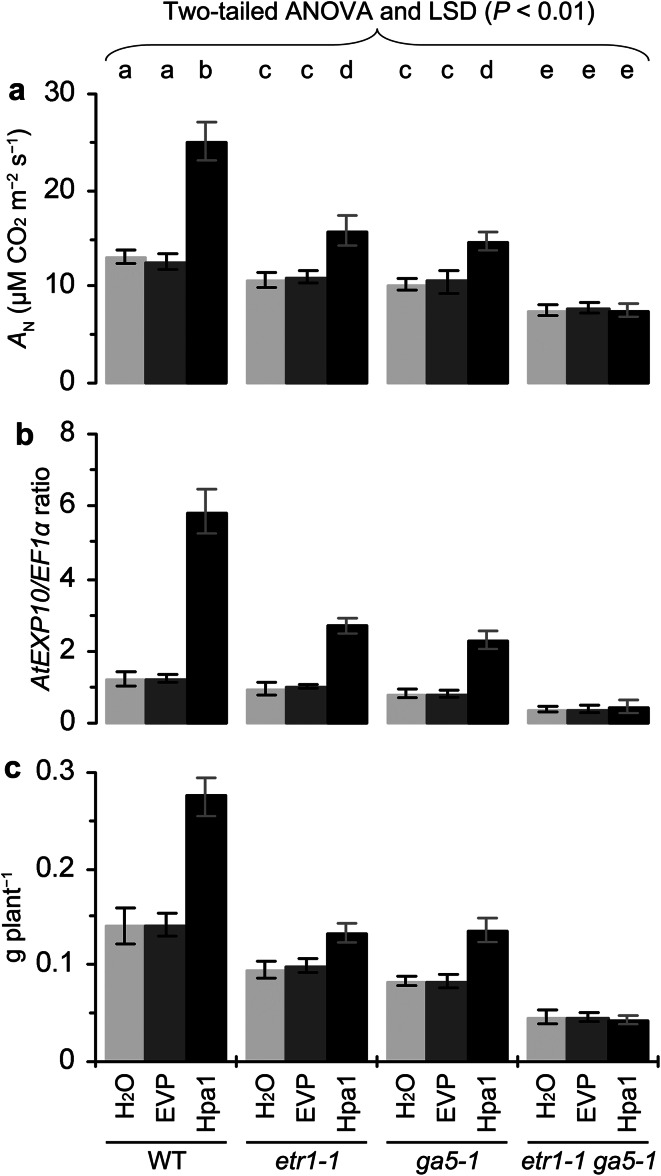
Genetic analysis for combinative effects of ethylene and gibberellin on Hpa1-enhanced photosynthesis, *EXP* expression, and growth of *Arabidopsis*. The WT plant, *etr1*-*1* and *ga5*-*1* single mutants, and *etr1*-*1 ga5*-*1* double mutant were tested in the experiments. Ten-day-old plants were treated separately with an aqueous Hpa1 solution and with pure water or an aqueous EVP solution. Five days later, analyses of *A*_N_ rates and *EXP* expression were performed on the second youngest leaves. Fresh weight of plants was scored 10 days after treatment. Data are shown as mean values ± SD from three experimental repeats (15 plants per repeat). *Different letters* on *bar* graphs indicate significant differences by the statistical analysis

To analyze whether ethylene and gibberellin coregulate Hpa1-enhanced photosynthesis, *EXP* expression, and plant growth, we generated the *Arabidopsis etr1*-*1 ga5*-*1* double mutant and characterized it based on nullified expression of both *ETR1* and *GA5* genes (Supplementary Fig. S3a), *etr1*-*1*-caused ethylene insensitivity in triple response (Supplementary Fig. S3b; Guzmán and Ecker 1990), and *ga5*-*1*-caused gibbrellin insensitivity in the silique development (Supplementary Fig. S3c; Vivian-Smith and Koltunow 1999). We compared the double mutant with the single mutants and the WT plant in terms of photosynthesis, *EXP* expression, and plant growth enhanced by Hpa1 compared to water or EVP. We confirmed that the promoting effects of Hpa1 on photosynthesis (Fig. 9a), *AtEXP10* expression (Fig. 9b), and plant growth (Fig. 9b) were partially eliminated in *etr1*-*1* or *ga5*-*1* compared to WT. We further found that all the effects of Hpa1 were canceled completely in *etr1*-*1 ga5*-*1*, which had similar levels of photosynthesis, *AtEXP10* expression, and growth irrespective of treatment with Hpa1 or Silwet-37 (Fig. 9a–c).

**Fig. 10:**
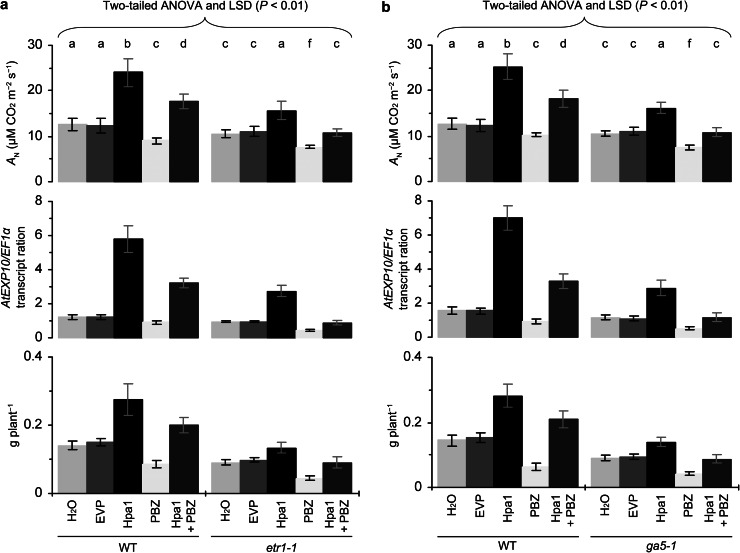
Genetic and chemical analyses for the interactive effects of ethylene and gibberellin on Hpa1-enhanced photosynthesis, *EXP* expression, and growth of *Arabidopsis.* **a** Ten-day-old WT and *etr1*-*1* plants were treated separately with pure water or an aqueous EVP solution and with aqueous solutions of Hpa1, PBZ, and a Hpa1 + PBZ mixture. **b** Ten-day-old WT and *ga5*-*1* plants were treated separately with pure water or an aqueous EVP solution and with aqueous solutions of Hpa1, 1-MCP, and a Hpa1 + 1-MCP mixture. **a**, **b** Five days after plant treatment, analyses of *A*_N_ rates and *EXP* expression were performed on the second youngest leaves. Fresh weight of plants was scored 10 days after treatment. Data shown are mean values ± SD from three experimental repeats (15 plants per repeat). *Different letters* on *bar* graphs indicate significant differences by the statistical analysis

We performed a genetic and chemical combinative analysis on WT and *etr1*-*1* plants by treating them separately with Hpa1, water, and EVP in the presence or absence of PBZ. This assay confirmed that the promoting effect of Hpa1 on photosynthesis, and plant growth were partially removed by the application of PBZ or in the *etr1*-*1* mutant compared to the WT plant (Fig. 10a). Noticeably, all the effects of Hpa1 were totally eliminated in the mutant following treatment with PBZ (Fig. 10a).

We assayed photosynthesis, *AtEXP10* expression, and growth of *ga5*-*1* and WT plants following treatment with Hpa1, water, or EVP in the presence and absence of 1-MCP, respectively. While the presence of 1-MCP diminished the effects of Hpa1 in the WT plant, 1-MCP applied to the *ga5*-*1* mutant totally eliminated all the effects of Hpa1 (Fig. 10b).

Taken together, these analyses indicate that ethylene and gibberellin indeed cooperate to regulate Hpa1-induced plant growth enhancement and the associated responses at physiological and molecular levels.

## Discussion

Harpin-induced plant growth enhancement was well demonstrated (Dong et al. 2004; Liu et al. 2006; Ren et al. 2006a, b, 2008; Oh and Beer 2007; Wu et al. 2007; Chen et al. 2008a, b), but the physiological and molecular mechanisms were not concomitantly elucidated. In this study, we utilize the purified Hpa1 protein (Fig. 1) as a model of harpins and characterize in detail the phenotype of Hpa1-induced plant growth enhancement (Fig. 2) in correlation with growth-promoting responses, including photosynthesis and related physiological responses (Figs. 3, 4), as well as the expression of *EXP* genes, especially *AtEXP10* and *LeEXP2* (Fig. 5; Supplementary Fig. S1) in *Arabidopsis*, tomato, and rice plants.

Substantial levels of *EXP* expression detected in 4 days after the plant treatment with the Hpa1 protein (Supplementary Fig. S1) indicate the stable effect of the protein. The stable effect of Hpa1 may be related to the stability of the protein in the plant. Harpin orthologs from different bacterial species (Choi et al. 2013) are conservatively sensitive to proteases (Wei et al. 1992; Kim and Beer 2000) and have the possibility of fast hydrolysis by plant proteases. If the hydrolysis occurs, it should impair the effects of harpins in plants. However, the relationship between the effects of harpins and their stability in plant was not directly elucidated in approximately 220 papers relating to harpins published since 1992. Based on the structural–functional relationship of harpins and their recognition by plant cells, we assume that the multiple effects of Hpa1 in plants (Peng et al. 2004; Liu et al. 2006; Ren et al. 2006a, b; Wu et al. 2007; Chen et al. 2008a; Sang et al. 2012) are guaranteed by three possible mechanisms.

Firstly, certain functional regions in the Hpa1 protein sequence (Liu et al. 2006; Wang et al. 2007, 2008; Chen et al. 2008a, b; Ji et al. 2011) allow its functions to perform in the case of hydrolysis within plant. If Hpa1 veritably undergoes a quick degradation, functional fragments of the protein are likely to resemble the full length in inducing plant growth enhancement and defense responses. The dual role of Hpa1 does not necessarily require full length of the protein, but instead, certain regions of the total protein sequence are more active experimentally (Wu et al. 2007; Chen et al. 2008a). For example, the 10–42-residue fragment (Hpa1_10–42_) isolated from the Hpa1 sequence is 1.3–7.5-fold more effective than the full length in inducing growth enhancement and defense responses in tea, tobacco, and rice as tested so far (Wu et al. 2007; Chen et al. 2008a, b). If Hpa1 veritably incurs a fast degradation in plant, the resulting fragments, such as Hpa1_10–42_ or containing Hpa1_10–42_ may be still active.

Secondly, harpin protein stability provides sufficient time for plant responses and harpin protein binding to plant cells (Oh and Beer 2007; Sang et al. 2012; Choi et al. 2013), probably reducing the possibility of hydrolysis. In this case, a stable activity for a certain period of time may be sufficient to induce plant responses. In a sole study about harpin protein stability, an unpurified HrpN_Ea_ preparation lost all bioactivity after 3 h at 37 °C or 6–8 h at 4 °C; and incubation of HrpN_Ea_ with protease type XIV at 100 μg mL^−1^, for 1 h at 37 °C destroyed bioactivity and eliminated the protein (Wei et al. 1992). Therefore, HrpN_Ea_ must be stable in plant for at least 1 h because the concentration of any proteases is impossible to be high as 100 μg mL^−1^ outside plant cells where harpins locate (Oh and Beer 2007; Sang et al. 2012; Choi et al. 2013), and because temperature in plant growth environment is unlikely to be high as 37 °C in most circumstances. The protein stability may be commonly shared by all harpin orthologs since they are conserved in biochemical characteristics (Wei et al. 1992; Kim and Beer 2000; Choi et al. 2013). If this hypothesis is correct, Hpa1 should be stable for at least 1 h in plant. This time is sufficient for plant responses (Fig. 6; Supplementary Figs. S1 and S2) even if harpin protein binding to plant cells (Oh and Beer 2007; Sang et al. 2012; Choi et al. 2013) does not increase the stability.

Thirdly, the activation of cellular signaling cascade determines duration of the effect of a harpin protein in plant (Dong et al. 1999, 2004, 2005). Plant sensing of Hpa1 and subsequent cellular responses are fast. Indeed, Hpa1 applied to *Arabidopsis* binds to the plasma membrane (Sang et al. 2012) and induces a rapid production of plant signaling compounds, such as hydrogen peroxide (H_2_O_2_), a cellular signal that mediates many aspects of plant development and defense responses (Nanda et al. 2010; Torres 2010; Sang et al. 2012). In Hpa1-treated *Arabidopsis*, H_2_O_2_ can be produced in the apoplast within 5 min and then the apoplastic H_2_O_2_ moves into the cytosol (Sang et al. 2012). The apoplastic production and cytosolic translocation of H_2_O_2_ are responsible for induced resistance to a bacterial pathogen in the plant (Sang et al. 2012). Similarly, the production of ethylene and gibberellin is highly induced within 12 h following the Hpa1 treatment (Fig. 6). Both hormones play an essential role in Hpa1-induced growth enhancement and the associated responses in the plant (Figs. 7, 8, 9, 10).

Previous studies show that ethylene plays a regulatory role in plant growth enhancement by HrpN_Ea_ (Dong et al. 2004; Ren et al. 2008), and the present study suggests that Hpa1 requires both ethylene and gibberellin to induce plant growth enhancement (Figs. 7, 8, 9, 10). We chose to elucidate the role of gibberellin in Hpa1-induced plant growth enhancement and the associated responses based on two considerations: (1) gibberellin is implicated in *EXP* expression associated with the vegetative growth of plants (Cho and Kende 1997; Vogler et al. 2003); (2) *EXP* expression is inducible by harpins (at least Hpa1 and HrpN_Ea_) and contributes to harpin-induced plant growth enhancement (Dong et al. 2004; Liu et al. 2006; Ren et al. 2006a, b; Wu et al. 2007; Chen et al. 2008a; Li et al. 2014). Meanwhile, we chose to study the cooperative roles of ethylene and gibberellin in the effects of Hpa1 on plants because of two reasons: (1) ethylene signaling was shown to be responsible for the phenotype of harpin-induced plant growth enhancement and the induction of *EXP* expression (Dong et al. 2004; Ren et al. 2008) but whether harpin-enhanced photosynthesis is also subject to ethylene signaling was unclear; (2) it is unclear whether gibberellin participates in the function of harpins in promoting photosynthesis and enhancing *EXP* expression.

Based on studies by genetic and chemical combinative methods, gibberellin and ethylene perform similarly in response to Hpa1 and individually plays a partial role in the regulation of Hpa1-enhanced photosynthesis, *EXP* expression, and plant growth (Figs. 7, 8). In other words, ethylene and gibberellin coregulate the full effects of Hpa1 in plants (Figs. 9, 10). Nevertheless, how both hormones are induced and how they regulate the functions of Hpa1 remain to be studied. The functional networks may be complicated. For instance, HrpN_Ea_ induces the protein kinase MPK6 (Desikan et al. 2001), and MPK6 phosphorylates 1-aminocyclopropane-1-carboxylic acid synthase (Liu and Zhang 2004), which is the rate-limiting enzyme and the major regulatory step in stress-induced ethylene production (Bleecker and Kende 2000; Wang et al. 2002). Moreover, photosynthesis relies on cellular CO_2_ uptake. On the basis of the significant role of CO_2_ transport across the plasma membrane (Heckwolf et al. 2011; Flexas et al. 2012; Kaldenhoff 2012; Uehlein et al. 2012), the phenotypic-physiological relevance for the Hpa1 activity may shed light on further characterization of whether and how Hpa1 is recognized by a protein located on the plasma membrane and of whether a molecular interaction is involved in the function of Hpa1. The postulated molecular interaction on the plasma membrane may be linked to the hormone signaling pathways as harpins (at least Hpa1 or HrpN_Ea_) are localized on the plasma membrane where they trigger a cellular signaling pathway to regulate plant defense responses or growth enhancement (Lee et al. 2001a, b; Oh and Beer 2007; Sang et al. 2012). Evidently, it is a great challenge to elucidate plant sensing of Hpa1 and subsequent responses that are connected to a cellular pathway of ethylene or gibberellin signal transduction.

The cooperation of ethylene and gibberellin may be specific for plant growth enhancement by a harpin protein since at least salicylic acid and jasmonic acid have been excluded from HrpN_Ea_-induced plant growth enhancement (Dong et al. 2004). However, there is as yet no evidence to exclude additional phytohormones, especially auxin, a phytohormone that is as multifunctional as ethylene or gibberellin and regulates many aspects of plant growth and development (Liscum and Reed 2002). Moreover, although harpin orthologs from different bacterial species have been shown to activate the same spectrum of hormone signaling (Strobel et al. 1996; Dong et al. 1999, 2004, 2005; Peng et al. 2004; Chen et al. 2008a; Zhang et al. 2011a, b; Sang et al. 2012), it is still necessary to study in the future whether the cooperative role of ethylene and gibberellin applies to different harpin orthologs in addition to Hpa1.

## Electronic supplementary material

Below is the link to the electronic supplementary material.

Supplementary material 1 (TIFF 9020 kb)

Supplementary material 2 (TIFF 3438 kb)

Supplementary material 3 (TIFF 5521 kb)

Supplementary material 4 (DOC 73 kb)

## Abbreviations

*A*_N_: Net photosynthesis (CO_2_ assimilation)
*ert1*-*1*: An *Arabidopsis* mutant with a defect in the ethylene receptor ETR1
EXP: Expansin
EVP: Empty vector preparation
*ga5*-*1*: An *Arabidopsis* mutant with a defect in the gibberellin biosynthesis
*etr1 ga5*-*1*: The double mutant
LSD: Fisher’s least significant difference
1-MCP: 1-Methylcyclopropene
PBZ: Paclobutrazol
RT-PCR: Reverse transcriptase-polymerase chain reaction

## References

[CR1] Alfano JR, Collmer A (2004). Type III secretion system effector proteins: double agents in bacterial disease and plant defense. Annu Rev Phytopathol.

[CR2] Beall FD, Morgan PW, Mander LN, Miller FR, Babb KH (1991). Genetic regulation of development in *Sorghum bicolor*. V. The *ma*_3_^R^ allele results in gibberellin enrichment. Plant Physiol.

[CR3] Bleecker AB, Kende H (2000). Ethylene: a gaseous signal molecule in plants. Annu Rev Cell Dev Biol.

[CR4] Chen L, Qian J, Qu SP, Long J, Yin Q, Zhang C, Wu X, Sun F, Wu T, Beer SV, Dong H (2008). Identification of specific fragments of HpaG_Xooc_, a harpin from *Xanthomonas oryzae* pv. *oryzicola*, that induce disease resistance and enhance growth in plants. Phytopathology.

[CR5] Chen L, Qian J, Qu SP, Yin Q, Qian J, Wu X, Sun F, Wu T, Cheng Z, Beer SV, Dong H (2008). A fragment of the *Xanthomonas oryzae* pv. *oryzicola* harpin HpaG_Xooc_ reduces disease and increases yield of rice in extensive grower plantings. Phytopathology.

[CR6] Cho HT, Cosgrove DJ (2002). Regulation of root hair initiation and expansin gene expression in *Arabidopsis*. Plant Cell.

[CR7] Cho HT, Kende H (1997). Expression of expansin genes is correlated with growth in deepwater rice. Plant Cell.

[CR8] Choi MS, Kim W, Lee C, Oh CS (2013). Harpins, multifunctional proteins secreted by gram-negative plant-pathogenic bacteria. Mol Plant-Microbe Interact.

[CR9] Colmer TD, Peeters AJ, Wagemaker CA, Vriezen WH, Ammerlaan A, Voesenek LA (2004). Expression of α-expansin genes during root acclimations to O_2_ deficiency in *Rumex palustris*. Plant Mol Biol.

[CR10] Cosgrove DJ (2001). Wall structure and wall loosening. A look backwards and forwards. Plant Physiol.

[CR11] Cox MC, Benschop JJ, Vreeburg RA, Wagemaker CA, Moritz T, Peeters AJ, Voesenek LA (2004). The roles of ethylene, auxin, abscisic acid, and gibberellin in the hyponastic growth of submerged *Rumex palustris* petioles. Plant Physiol.

[CR12] Desikan R, Hancock JT, Ichimura K, Shinozaki K, Neill SJ (2001). Harpin induced activation of the *Arabidopsis* mitogen-activated protein kinases AtMPK4 and AtMPK6. Plant Physiol.

[CR13] Ding X, Cao Y, Huang L, Zhao J, Xu C, Li X, Wang S (2008). Activation of the indole-3-acetic acid-amido synthetase GH3-8 suppresses expansin expression and promotes salicylate- and jasmonate-independent basal immunity in rice. Plant Cell.

[CR14] Dong HS, Delaney TP, Bauer DW, Beer SV (1999). Harpin induces disease resistance in *Arabidopsis* through the systemic acquired resistance pathway mediated by salicylic acid and the *NIM1* gene. Plant J.

[CR15] Dong HP, Peng JL, Bao ZL, Meng XD, Bonasera JM, Chen GY, Beer SV, Dong HS (2004). Downstream divergence of ethylene signaling pathway for harpin-stimulated *Arabidopsis* growth and insect defense. Plant Physiol.

[CR16] Dong HP, Yu HQ, Bao ZL, Guo XJ, Peng JL, Yao Z, Chen GY, Qu SP, Dong HS (2005). The *ABI2*-dependent abscisic acid signalling controls HrpN-induced drought tolerance in *Arabidopsis*. Planta.

[CR17] Flexas J, Ortuño MF, Ribas-Carbo M, Diaz-Espejo A, Flórez-Sarasa ID, Medrano H (2007). Mesophyll conductance to CO_2_ in *Arabidopsis thaliana*. New Phytol.

[CR18] Flexas J, Barbour MM, Brendel O, Cabrera HM, Carriquí M, Díaz-Espejo A, Douthe C, Dreyer E, Ferrio JP, Gago J, Gallé A, Galmés J, Kodama N, Medrano H, Niinemets Ü, Peguero-Pina JJ, Pou A, Ribas-Carbó M, Tomás M, Tosens T, Warren CR (2012). Mesophyll diffusion conductance to CO_2_: an unappreciated central player in photosynthesis. Plant Sci.

[CR19] Foster KR, Miller FR, Childs KL, Morgan PW (1994). Genetic regulation of development in *Sorghum bicolor*. VIII. Shoot growth, tillering, flowering, gibberellin biosynthesis, and phytochrome levels are differentially affected by dosage of the *ma*_3_^R^ allele. Plant Physiol.

[CR20] Gamble RL, Qu X, Schaller GE (2002). Mutational analysis of the ethylene receptor ETR1. Role of the histidine kinase domain in dominant ethylene insensitivity. Plant Physiol.

[CR21] Guzmán P, Ecker JR (1990). Exploiting the triple response of *Arabidopsis* to identify ethylene-related mutants. Plant Cell.

[CR22] He SY, Huang HC, Collmer H (1993). *Pseudomonas syringae* pv. syringae harpin_pss_: a protein that is secreted via the hrp pathway and elicits the hypersensitive response in plants. Cell.

[CR23] Heckwolf M, Pater D, Hanson DT, Kaldenhoff R (2011). The *Arabidopsis thaliana* aquaporin AtPIP1;2 is a physiologically relevant CO_2_ transport facilitator. Plant J.

[CR24] Ji Z, Song C, Lu X, Wang J (2011). Two coiled-coil regions of *Xanthomonas oryzae* pv. *oryzae* harpin differ in oligomerization and hypersensitive response induction. Amino Acids.

[CR25] Kaldenhoff R (2012). Mechanisms underlying CO_2_ diffusion in leaves. Curr Opin Plant Biol.

[CR26] Kim JF, Beer SV, Vanneste JL (2000). *Hrp* genes and Harpins of *Erwinia amylovora*: a decade of discovery. Fire blight and its causative agent *Erwinia amylovora*.

[CR27] Kourill R, Ilik P, Naus J, Schoefs B (1999). On the limits of applicability of spectrophotometric and spectrofluorimetric methods for the determination of chlorophyll a/b ratio. Photosyn Res.

[CR28] Lang CA (1958). Simple micro-determination of Kjeldahl nitrogen in biological materials. Anal Chem.

[CR29] Lee J, Klessig DF, Nurnberger T (2001). A harpin binding site in tobacco plasma membranes mediates activation of the pathogenesis-related gene *HIN1* independent of extracellular calcium but dependent on mitogen-activated protein kinase activity. Plant Cell.

[CR30] Lee J, Klusener B, Tsiamis G, Stevens C, Neyt C, Tampakaki AP, Panopoulos NJ, Noller J, Weiler EW, Cornelis GR, Mansfield JW, Nürnberger T (2001). HrpZ_Psph_ from the plant pathogen *Pseudomonas syringae* pv. *phaseolicola* binds to lipid bilayers and forms an ion-conducting pore in vitro. Proc Natl Acad Sci USA.

[CR32] Li XJ, Zhao YY, You ZZ, Dong HS, Zhang CL (2014) The Hpa1 harpin needs nitroxyl terminus to promote vegetative growth and leaf photosynthesis in *Arabidopsis.* J Biosci 39 (in press) 10.1007/s12038-013-9408-6 24499797

[CR33] Liscum E, Reed JW (2002). Genetics of Aux/IAA and ARF action in plant growth and development. Plant Mol Biol.

[CR34] Liu YD, Zhang SQ (2004). Phosphorylation of 1-aminocyclopropane-1-carboxylic acid synthase by MPK6, a stress-responsive mitogen-activated protein kinase, induces ethylene biosynthesis in *Arabidopsis*. Plant Cell.

[CR35] Liu F, Liu H, Jia Q, Wu X, Guo X, Zhang S, Song F, Dong H (2006). The internal glycine-rich motif and cysteine suppress several effects of the HpaG_Xooc_ protein in plants. Phytopathology.

[CR36] Liu RX, Chen L, Jia ZH, Lü BB, Shi HJ, Shao W, Dong HS (2011). Transcription factor AtMYB44 regulates induced expression of the *ETHYLENE INSENSITIVE2* gene in *Arabidopsis* responding to a harpin protein. Mol Plant-Microbe Interact.

[CR37] Lü BB, Li XJ, Sun WW, Li L, Gao R, Zhu Q, Tian SM, Fu MQ, Yu HL, Tang XM, Zhang CL, Dong HS (2013). AtMYB44 regulates resistance to the green peach aphid and diamondback moth by activating *EIN2*-affected defences in *Arabidopsis*. Plant Biol (Stuttg).

[CR38] Lü P, Kang M, Jiang X, Dai F, Gao J, Zhang C (2013). *RhEXPA4*, a rose expansin gene, modulates leaf growth and confers drought and salt tolerance to *Arabidopsis*. Planta.

[CR39] Miao WG, Wang XB, Li M, Song CF, Wang Y, Hu DW, Wang JS (2010). Genetic transformation of cotton with a harpin-encoding gene *hpa*_*Xoo*_ confers an enhanced defense response against different pathogens through a priming mechanism. BMC Plant Biol.

[CR40] Nanda AK, Andrio E, Marino D, Pauly N, Dunand C (2010). Reactive oxygen species during plant-microorganism early interactions. J Integr Plant Biol.

[CR41] Oh CS, Beer SV (2007). AtHIPM, an ortholog of the apple HrpN-interacting protein, is a negative regulator of plant growth and mediates the growth-enhancing effector of HrpN in Arabidopsis. Plant Physiol.

[CR42] Olmedo G, Guo HW, Gregory BD, Nourizadeh SD, Aguilar- Henonin L, Li HJ, An FY, Guzman P, Ecker JR (2006). *Ethylene*-*insensitive5* encodes a 5′ → 3′ exoribonuclease required for regulation of the EIN3-targeting F-box proteins EBF1/2. Proc Natl Acad Sci USA.

[CR43] Peng JL, Bao ZL, Ren HY, Wang JS, Dong HS (2004). Expression of harpin_Xoo_ in transgenic tobacco induces pathogen defense in the absence of hypersensitive cell death. Phytopathology.

[CR44] Penninckx IA, Thomma BP, Buchala A, Metraux JP, Broekaert WF (1998). Concomitant activation of jasmonate and ethylene response pathways is required for induction of a plant defensin gene in Arabidopsis. Plant Cell.

[CR45] Pieterse CM, van Wees SC, van Pelt JA, Knoester M, Laan R, Gerrits H, Weisbeek PJ, van Loon LC (1998). A novel signaling pathway controlling induced systemic resistance in *Arabidopsis*. Plant Cell.

[CR46] Potuschak T, Vansiri A, Binder BM, Lechner E, Vierstra RD, Genschik P (2006). The exoribonuclease XRN4 is a component of the ethylene response pathway in *Arabidopsis*. Plant Cell.

[CR47] Ren HY, Gu GY, Long JY, Wu TQ, Song T, Zhang SJ, Chen ZY, Dong HS (2006). Combinative effects of a bacterial type-III effector and a biocontrol bacterium on rice growth and disease resistance. J Biosci.

[CR48] Ren HY, Song T, Wu TQ, Sun L, Liu YX, Yang FF, Chen ZY, Dong HS (2006). Effects of a biocontrol bacterium on growth and defence of transgenic rice plants expressing a bacterial type-III effector. Annals Microbiol.

[CR49] Ren XY, Liu F, Bao ZL, Zhang CL, Wu X, Chen L, Liu RX, Dong HS (2008). Root growth of *Arabidopsis thaliana* is regulated by ethylene and abscisic acid signaling interaction in response to HrpN_Ea_, a bacterial protein of harpin group. Plant Mol Biol Rep.

[CR50] Rose JKC, Bennett AB (1999). Cooperative disassembly of the cellulose-xyloglucan network of plant cell walls: parallels between cell expansion and fruit ripening. Trends Plant Sci.

[CR51] Sampedro J, Cosgrove DJ (2005). The expansin superfamily. Genome Biol.

[CR52] Sang S, Li X, Gao R, You Z, Lü B, Liu P, Ma Q, Dong H (2012). Apoplastic and cytoplasmic location of harpin protein Hpa1_Xoo_ plays different roles in H_2_O_2_ generation and pathogen resistance in *Arabidopsis*. Plant Mol Biol.

[CR53] Schaller GE, Bleecker AB (1995). Ethylene-binding sites generated in yeast expressing the *Arabidopsis**ETR1* gene. Science.

[CR111] Shi LW (2012) SPSS19.0 Statistical analysis from accidence to conversance (in Chinese). Tsinghua University Press, Beijing, pp 109–143

[CR54] Sloan J, Backhaus A, Malinowski R, McQueen-Mason S, Fleming AJ (2009). Phased control of expansin activity during leaf development identifies a sensitivity window for expansin-mediated induction of leaf growth. Plant Physiol.

[CR55] Soltys D, Rudzińska-Langwald A, Gniazdowska A, Wiśniewska A, Bogatek R (2012). Inhibition of tomato (*Solanum lycopersicum* L.) root growth by cyanamide is due to altered cell division, phytohormone balance and expansin gene expression. Planta.

[CR56] Sponsel VM, Schmidt FW, Porter SG, Nakayama M, Kohlstruk S, Estelle M (1997). Characterization of new gibberellin-responsive semidwarf mutants of *Arabidopsis*. Plant Physiol.

[CR57] Strobel RN, Gopalan JS, Kuc JA, He SY (1996). Induction of systemic acquired resistance in cucumber by *Pseudomonas syringae* pv. *syringae* 61 HrpZ_Pss_ protein. Plant J.

[CR58] Torres MA (2010). ROS in biotic interactions. Plant Physiol.

[CR59] Uehlein N, Sperling H, Heckwolf M, Kaldenhoff R (2012). The *Arabidopsis* aquaporin PIP1;2 rules cellular CO_2_ uptake. Plant Cell Environ.

[CR60] Vivian-Smith A, Koltunow AM (1999). Genetic analysis of growth-regulator-induced parthenocarpy in *Arabidopsis*. Plant Physiol.

[CR61] Vogler H, Caderas D, Mandel T, Kuhlemeier C (2003). Domains of expansin gene expression define growth regions in the shoot apex of tomato. Plant Mol Biol.

[CR62] Wang KL, Li H, Ecker JR (2002). Ethylene biosynthesis and signaling networks. Plant Cell.

[CR63] Wang X, Li M, Zhang J, Zhang Y, Zhang G, Wang J (2007). Identification of a key functional region in harpins from *Xanthomonas* that suppresses protein aggregation and mediates harpin expression in *E. coli*. Mol Biol Rep.

[CR64] Wang X, Song C, Miao W, Ji Z, Wang B, Zhang Y, Zhang J, Hu JS, Borth W, Wang J (2008). Mutations in the N-terminal coding region of the harpin protein Hpa1 from *Xanthomonas oryzae* cause loss of hypersensitive reaction induction in tobacco. Appl Microbiol Biotechnol.

[CR65] Wang G, Gao Y, Wang J, Yang L, Song R, Li X, Shi J (2011). Overexpression of two cambium-abundant Chinese fir (*Cunninghamia lanceolata*) α-expansin genes *ClEXPA1* and *ClEXPA2* affect growth and development in transgenic tobacco and increase the amount of cellulose in stem cell walls. Plant Biotechnol J.

[CR66] Wei ZM, Laby RJ, Zumoff CH, Bauer DW, He SY, Collmer A, Beer SV (1992). Harpin, elicitor of the hypersensitive response produced by the plant pathogen *Erwinia amylovora*. Science.

[CR67] Wu XJ, Wu TQ, Long JY, Yin Q, Zhang Y, Chen L, Liang Y, Liu RX, Gao TC, Dong HS (2007). Productivity and biochemical properties of green tea in response to a bacterial type-III effector protein and its variants. J Biosci.

[CR68] Xu YL, Li L, Wu K, Peeters AJ, Gage DA, Zeevaart JA (1995). The *GA5* locus of *Arabidopsis thaliana* encodes a multifunctional gibberellin 20-oxidase: molecular cloning and functional expression. Proc Natl Acad Sci USA.

[CR69] Yang QH, Lu W, Hu ML, Wang CM, Zhang RX, Yao M, Wan JM (2003). QTL and epistatic interaction underlying leaf chlorophyll and H_2_O_2_ content variation in rice (*Oryza sativa* L.). Yi Chuan Xue Bao.

[CR70] Zhang CL, Bao ZL, Liang Y, Yang X, Wu X, Hong X, Dong HS (2007). Abscisic acid mediates *Arabidopsis* drought tolerance induced by HrpN_Ea_ in the absence of ethylene signaling. Plant Mol Biol Rep.

[CR71] Zhang CL, Shi HJ, Chen L, Wang XM, Lü BB, Zhang SP, Liang YA, Liu RX, Qian J, Sun WW, You ZZ, Dong HS (2011). Harpin-induced expression and transgenic overexpression of the phloem protein gene *AtPP2*-*A1* in *Arabidopsis* repress phloem feeding of the green peach aphid *Myzus persicae*. BMC Plant Biol.

[CR72] Zhang L, Xiao S, Li W, Feng W, Li J, Wu Z, Gao X, Liu F, Shao M (2011). Overexpression of a Harpin-encoding gene *hrf1* in rice enhances drought tolerance. J Exp Bot.

[CR73] Zhao MR, Han YY, Feng YN, Li F, Wang W (2012). Expansins are involved in cell growth mediated by abscisic acid and indole-3-acetic acid under drought stress in wheat. Plant Cell Rep.

[CR74] Zhu WG, Magbanua MM, White FF (2000). Identification of two novel *hpaG*-associated genes in the *hpaG* gene cluster of *Xanthomonas oryzae* pv. *oryzae*. J Bacteriol.

