# Supplementary figures and images for: Plant growth enhancement and associated physiological responses are coregulated by ethylene and gibberellin in response to harpin protein Hpa1
Source: Planta. 2014 Jan 7;239(4):831–46. doi: 10.1007/s00425-013-2013-y (PMC3955481; doi:10.1007/s00425-013-2013-y)

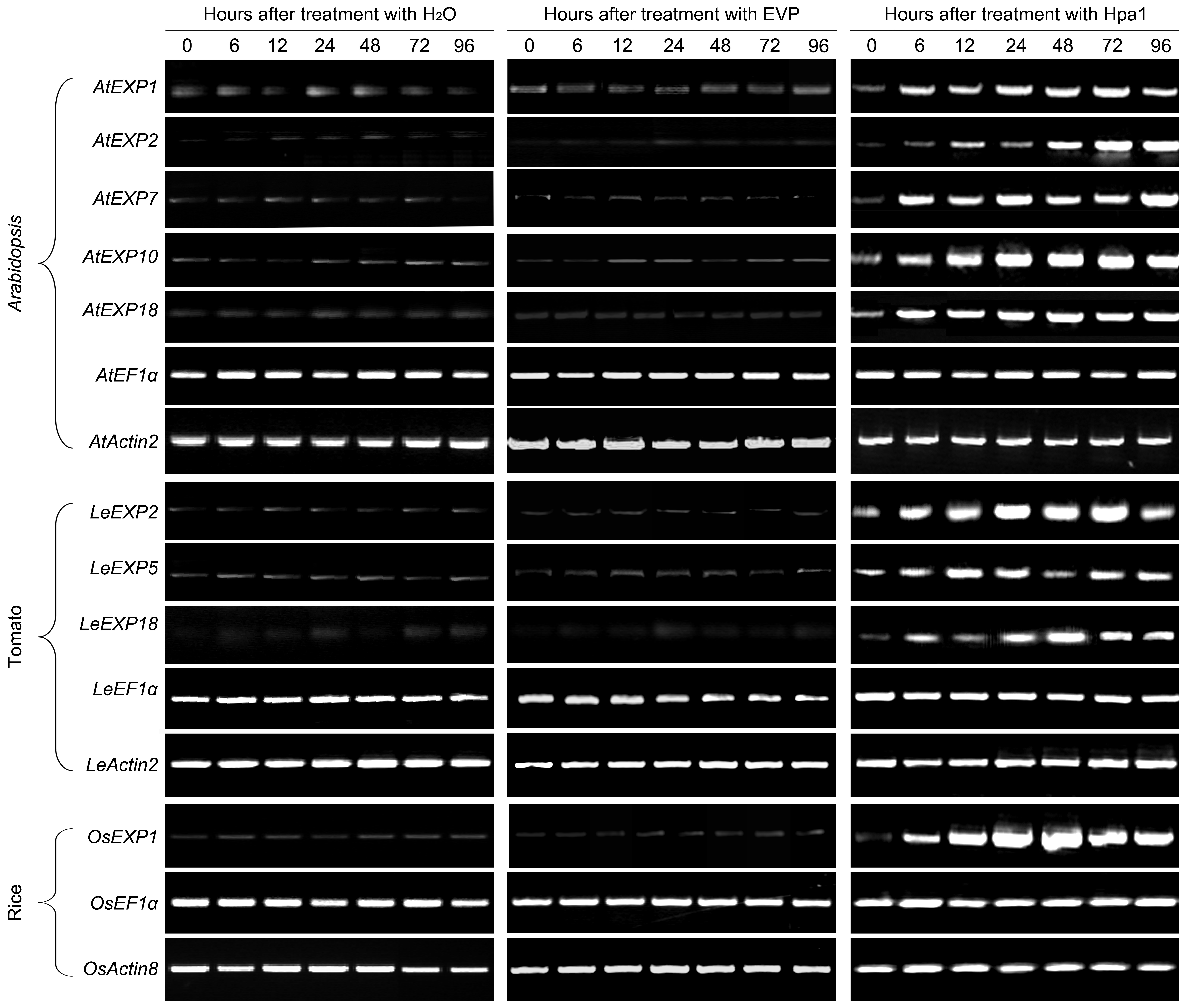

Supplement: Supplementary file 1 — Supplementary material 1 (TIFF 9020 kb) [file 425_2013_2013_MOESM1_ESM.tif]

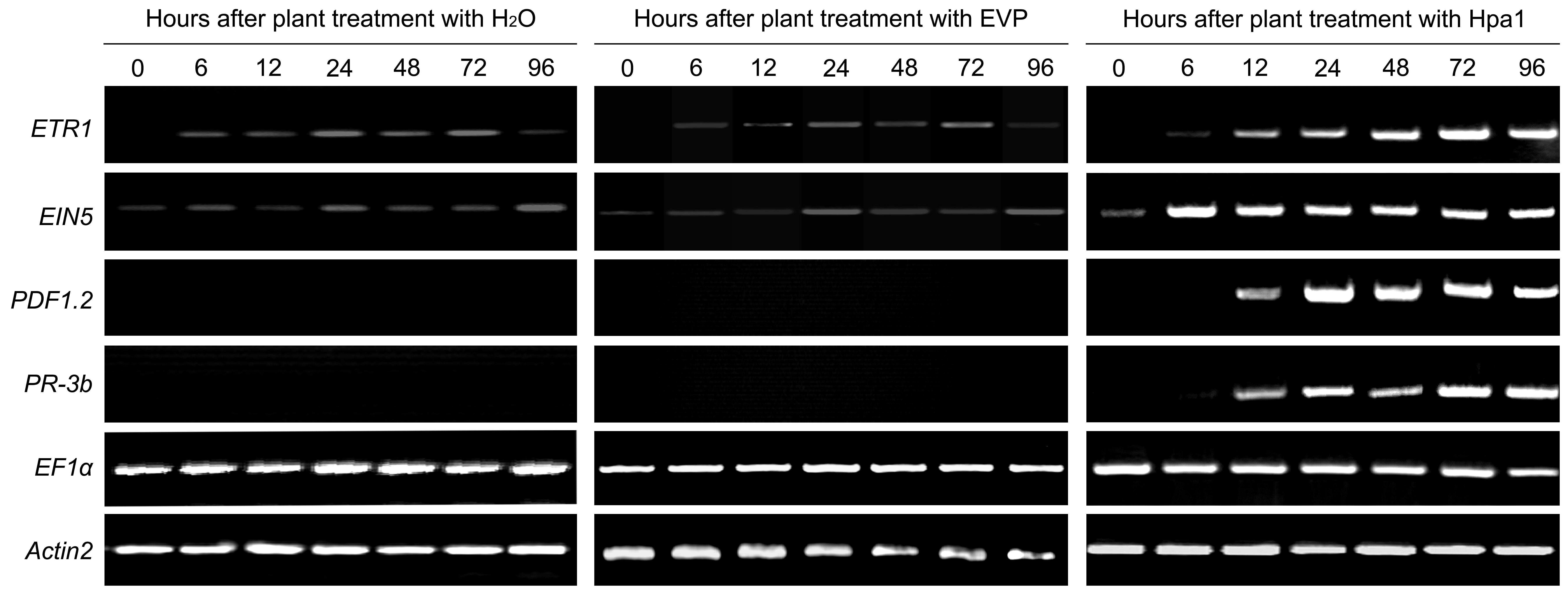

Supplement: Supplementary file 2 — Supplementary material 2 (TIFF 3438 kb) [file 425_2013_2013_MOESM2_ESM.tif]

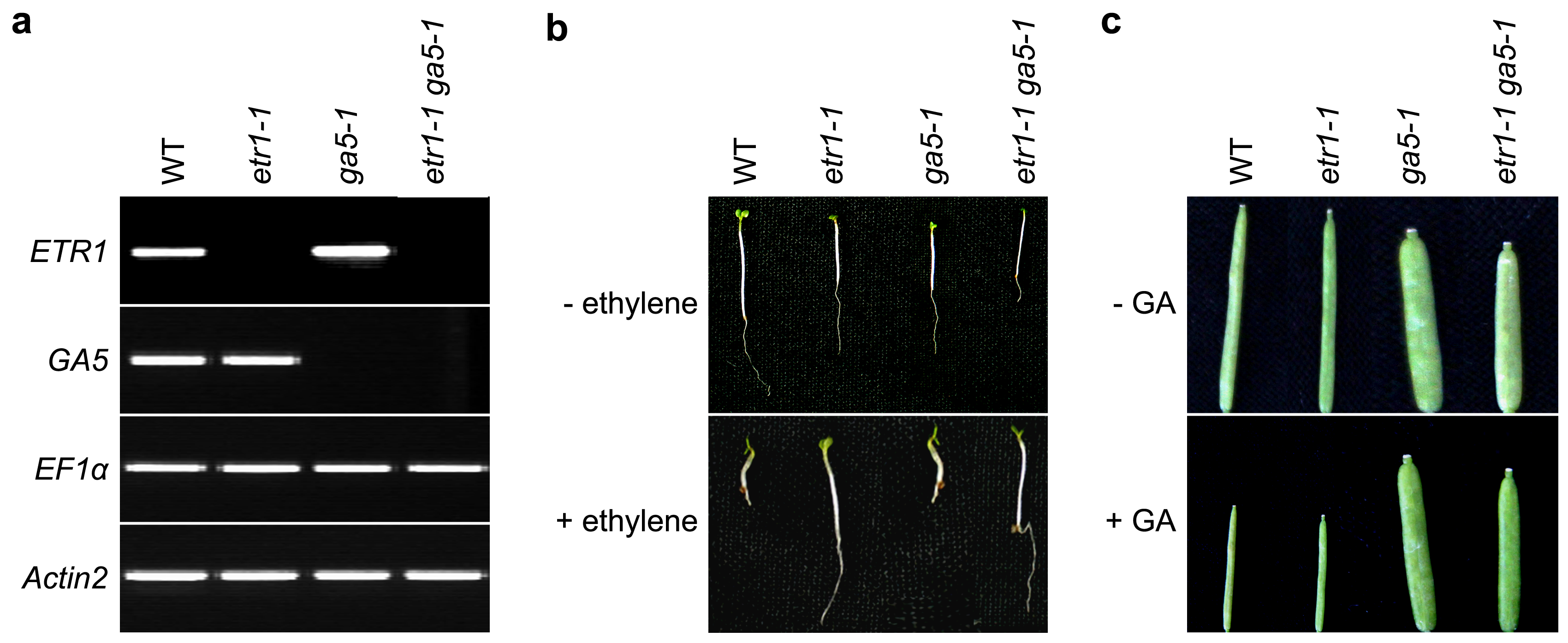

Supplement: Supplementary file 3 — Supplementary material 3 (TIFF 5521 kb) [file 425_2013_2013_MOESM3_ESM.tif]
