# Supplementary material for: Plant growth enhancement and associated physiological responses are coregulated by ethylene and gibberellin in response to harpin protein Hpa1
Source: Planta. 2014 Jan 7;239(4):831–46. doi: 10.1007/s00425-013-2013-y (PMC3955481; doi:10.1007/s00425-013-2013-y)
Supplement: Supplementary file 4 — Supplementary material 4 (DOC 73 kb) [file 425_2013_2013_MOESM4_ESM.doc]

Li X et al

**Plant growth enhancement and associated physiological responses are coregulated by ethylene and gibberellin in response to harpin protein Hpa1**

**Supplementary Material**

**Supplementary Fig. S1** A four-day profile of *EXP* gene expression in *Arabidopsis*, tomato, and rice leaves. Ten-day-old plants were treated as shown on top panel. RNA was isolated from leaves at the indicated time points and analyzed by reverse polymerase chain reaction (RT-PCR) using the constitutively expressed *EF1α* and *Actin* genes as references

**Supplementary Fig. S2** A four-day profile of the expression of ethylene signaling and responsive genes in *Arabidopsis* leaves. Ten-day-old plants were treated as shown on top panel. RNA was isolated from leaves at the indicated time points and analyzed by RT-PCR using *EF1α* and *Actin* genes as references

**Supplementary Fig. S3** Characterization of the *etr1-1 ga5-1* double mutant compared to the WT plant and single mutants. **a**, RT-PCR analyses of gene expression in the indicated plants. **b**, Triple response to ethylene (+ ethylene) in contrast to the normal growth of seedlings in the absence of ethylene (- ethylene). Ethylene was converted from the precursor 1-aminocyclopropane-1-carboxylate applied to germinating seeds in a sealed glass container in which the ethylene amount was 20 μl (Liu et al. 2011). **c**, Early silique development in the absence (-) and presence (+) of the pistil treatment with 10 μM gibberellin GA3 ([Vivian-Smith and](http://www.ncbi.nlm.nih.gov/pubmed?term=Vivian-Smith A%5BAuthor%5D&cauthor=true&cauthor_uid=10517835) [Koltunow 1999](http://www.ncbi.nlm.nih.gov/pubmed?term=Koltunow AM%5BAuthor%5D&cauthor=true&cauthor_uid=10517835))

**Supplementary Table 1** Information on genes tested and primers used in this study

| Plant | Gene | Locus code | Primers* (product size in bp) |
| --- | --- | --- | --- |
| *Arabidopsis* | ETR1 | L24119 | 5’-GGAATTCCATATGGAAGTCTGCAATTGTATTGAACC-3’, 5’-CGGGATCCTTACTCTCTAAATAATGTATGAAGATTGA-3’; (1284) |
|  | GA5 | ATU20873 | 5’-CTTTGATATGCCTCTCTCCG-3’, 5’-TTTCCTCATCCTCTCGCTGT-3’; (598) |
|  | EIN5 | BT026022 | 5’-GTCGCTCTTCAGTATTACATCC-3’, 5’-TTATCGTCTATCGGTTCAGGT-3’; (791) |
|  | PDF1.2 | T04323 | 5’-AGAAATATGCATGTCATAAAGTTACTCAT-3’, 5’-CAATGGTGGAAGCACAGAAG-3’; (244) |
|  | PR-3b | AB023463 | 5’-CTACAGCACCAGACGGACCATA-3’, 5’-CTAAATAGCAGCTTCGAGGAGGCC-3’; (539) |
|  | AtEXP1 | NM_179537 | 5’-ACATTCTACGGTGGTGGTGATGC-3’, 5’-CACTCTTCCTAACGTAGCTGCGC-3’; (665) |
|  | *AtEXP2* | NM_120611 | 5’-ACGGTAACTTACACAGCCAAGGC-3’, 5’-GCACAACATCGTAGCTCACAACAG-3’; (557) |
|  | *AtEXP7* | NM_101127 | 5’-CATGGAGATATGCTCACGCCAC-3’, 5’-GCTTATCCAATTCGTCCGGCTA-3’ (512) |
|  | *AtEXP10* | AF229437 | 5’-CCGCTACAAACTTCTGCCCGCCAAAT-3’, 5’-ACCGAGCCCTCGACGCATCACTCT-3’ (574) |
|  |  |  | 5’-GCTATGTCAAGGAACTGGGGGC-3’, 5’-CAGGAGCGGCGTTGAAGGAGAC-3’; (121) |
|  | *AtEXP18* | NM_104976 | 5’-GGCCTTCTGTGTTGCTTCACT-3’, 5’-TATGGAGGAACATAGGCATGGC-3’ (413) |
|  | *AtEF1α* | AY123029 | 5’-AGACCACCAAGTACTACTGCAC-3’, 5’-CCACCAATCTTGTACACATCC-3’; (495) |
|  |  |  | 5’-ATCCCATTTGTGCCCATCTCTG-3’,  5’-CTTGTAGACATCCTGAAGTGGG-3’; (168) |
|  | *AtActin2* | AY096381 | 5’-GGATTCTGGTGATGGTGTGTC-3’, 5’-TGCAAGTGCTGTGATTTCTTT-3’; (505) |
| Tomato | LeEXP2 | AF096776 | 5’-ACACTGCAGCACTAAGTACAGC-3’, 5’-AAATTACAAGTTGAAAACCCCTC-3’ (616) |
|  |  |  | 5’-TAGCCAAGGGTATGGAACTAAC-3’, 5’-TTGTTAGGTAGAGACGGGTTCG-3’ (177) |
|  | LeEXP5 | AF059489 | 5’-GCGTTGAGTACTGCTCTGTTC-3’, 5’-TACTTCCACAATTGAATGACC-3’; (718) |
|  | *LeEXP18* | LEAJ4997 | 5’-GGCCTTCTGTGTTGCTTCACT-3’, 5’-TATGGAGGAACATAGGCATGGC-3’ (413) |
|  | *LeEF1α* | NM_001247106 | 5’-TACAACCCTGACAAAATCCCCT-3’, 5’-CCTTCTCAAGTTCCTTACCTGA-3’; (586) |
|  |  |  | 5’-CTGTTCTCATTATTGACTCCAC-3’, 5’-GGGGGTGGTAGCATCCATCTTG-3’; (146) |
|  | *LeActin2* | XM_004231715 | 5’-TCATTTCCTCACCTCACGAAA-3’, 5’-ATCCAAAACTCAGGGGCATCC-3’; (599) |
| Rice | *OsEXP1* | NM_001058824 | 5’-CGTCGTGTGCTCGGTTCCTG-3’, 5’-ACCTCACCCCGCCCTGCTTG-3’; (486) |
|  |  |  | 5’-CGTCGTGTGCTCGGTTCCT-3’, 5’-GCCCCACCCATCGTTCCTG-3’ (139) |
|  | *OsEF1α* | AF030517 | 5’-ATGGGTAAGGAGAAGACGC-3’, 5’-CAGAGATGGGAACAAAGGG-3’;(583) |
|  |  |  | 5’-CGTGCCTGTGGGTCGTGTTG-3’, 5’-TCCTGGAGAGCCTCGTGGTG-3’; (120) |
|  | *OsActin8* | AY212324 | 5’-ACTCCTTCACTACCACTGC-3’, 5’-CCCGACTCATCATACTCTC-3’; (505) |

*Primers for shorter products were used in real-time reverse transcriptase-polymerase chain reaction (RT-PCR), and others were used in RT-PCR.
